# Recent advances and effective strategies in the discovery and applications of natural products

**DOI:** 10.1039/c7ra09475b

**Published:** 2018-01-03

**Authors:** Jing Xie, Ai-hua Zhang, Hui Sun, Guang-li Yan, Xi-jun Wang

**Affiliations:** Sino-America Chinmedomics Technology Collaboration Center, National TCM Key Laboratory of Serum Pharmacochemistry, Metabolomics Laboratory, Department of Pharmaceutical Analysis, Heilongjiang University of Chinese Medicine Heping Road 24 Harbin 150040 China xijunwangtcm@126.com +86-451-82110818 +86-451-82110818

## Abstract

Natural products are the most representative form of conventional therapy as compared to any other traditional or alternative medicine systems. They have numerous active components, either primary or secondary metabolites, which are associated with the diverse, intricate, and distinct characteristics of natural products and result in various pharmacological effects in clinic. However, some problems are associated with research on herb quality, which is the core of the drug industry, and restrict the development of this field to a certain extent. Quality-markers (Q-markers), a novel concept for quality assessment, open up a new avenue for promotion of healthy development of traditional medicine industry and improvement of the quality standard system to enhance traditional medicine or product quality standards. In this study, we first summarized the main factors affecting the quality of traditional medicines and natural products and importance for safety and then presented the concept of background and relevant factors of Q-markers. Moreover, the modern science technology and related methods used to identify the chemical composition have been discussed. Especially, based on the systematic analysis and discussion of the basic properties and clinical features of natural products, we have discussed new trends and effective strategies for identifying relevant Q-markers from herbs and probed the future research directions and challenges.

## Abstract 2

Natural products are the most representative form of conventional therapy as compared to any other traditional or alternative medicine systems.

## Graphical abstract

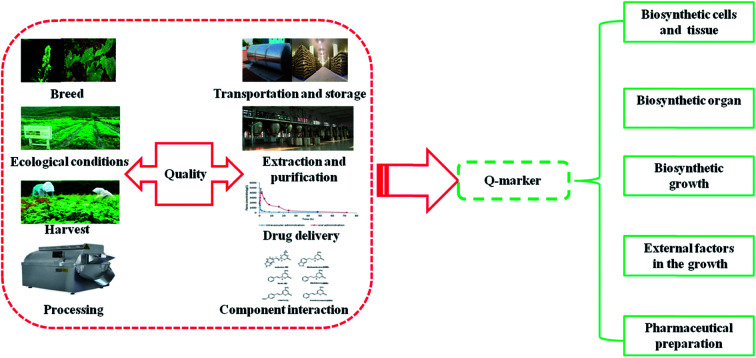

## 1. Introduction

With traditional medicines entering a worldwide new arena in clinical medicine and pharmaceutical research, natural products are becoming popular all over the world and are being widely accepted as a conventional clinic therapy as compared to the currently available therapeutic options.^1,2^ To date, a number of studies have been focused on herbs that play a major role in health promotion as well as prevention, management, and treatment of various diseases due to their effectiveness, convenience, fewer side effects, and relatively low cost.^3,4^ Traditional medicines have complications such as multiple sources and production area, leading to an obvious difference in their quality, especially the number of effective ingredients is significantly different;^5^ it is worth noting that the quality of herbs determines the effectiveness and safety of herbs in traditional medicines.^6^ Increasing public awareness and scientific interest have spurred research towards the establishment of the quality of traditional medicine research and quality standards; this is complex system engineering due to involvement of different methods and different opinions. Drug evaluation standard is the basic standard of drug quality.^7^ By establishing qualitative and quantitative detection methods to obtain some feature analysis methods and fingerprints, it has been found that traditional medicines exhibit specificity and controllability, which are the basis of quality standards.^8^ The system of quality standards recognized by industry that uses one or several index component contents to assess traditional medicines is under study and controversial. However, the clinical efficacy of traditional medicine is not necessarily related to the role of a detected indicator component.^9^

To overcome this obstacle, improvement of consistency, controllability, and originality of quality is conducive to the control and quality supervision of traditional medicines in the production process. Professor Liu has recently put forward a new concept for identifying relevant quality-markers (Q-markers) from herbs that aid in building the quality standards for herbs and corresponding products.^10^ Utilizing the concept of Q-markers, well-known experts and scholars have also put forward research ideas and methods such as chinmedomics, property–response–component mode, efficacy–syndrome–toxicity mode, as well as effect-constituent index. In this study, we have initially presented the essential role of quality of traditional medicines as the core of validity and safety in medical research, and then we have introduced the definition, impact factor, and significance of Q-markers in quality assessment. We especially focused on discussing new trends and effective methodology and technology for identifying relevant Q-markers from herbs. Finally, we have discussed the future research directions and challenges.

## 2. Quality of traditional medicines: the core of drug safety and efficacy

Herbal drugs have been accepted in the framework of medicinal products in the European market for a long time, varying from country to country;^11^ measures are taken to prove the quality, efficacy, and safety of herbal drugs and products before their access to the market just as all other medicines.^12^ Quality of traditional medicines, affected by many factors such as variety, cultivation, processing, transportation, and storage supervision, is the basis for ensuring the efficacy and safety of drugs in clinical use.^13^ If the quality of the herbal medicine is not qualified, for example, the effective ingredient does not meet the standards or contain toxic components, it will affect the clinical therapeutic effect and the adverse reaction events caused by the natural drug. Authenticity, advantages and disadvantages play a vital role in clinical efficacy, and some even endanger the lives of people.^14,15^ Due to the lack of standardized management and technical guidance, planting techniques lead to poor germplasm, degradation of varieties, and other issues. It is worth noting that the harvest of traditional medicine is usually based on quality optimization to the greatest extent and yield maximization of herbs, but the two aspects cannot be achieved at the same time. Therefore, selection of an appropriate harvesting period and processing method to receive better quality of medicine is urgently needed for clinical efficacy.^16^ The purpose of traditional medicine processing includes attenuation, efficiency, synergy, and component extraction, which directly affect the effectiveness and amount of the active substance. Traditional medicines in the storage and transportation process are vulnerable to air, temperature, humidity, light, and other effects, leading to complicated physical and chemical changes.^17^ The extraction and purification process of traditional medicines is the key to quality transmission of the drug. From the perspective of safety and effectiveness of drugs to understand and evaluate the quality of traditional medicines, not only evaluation of the prototype components is conducted, but also analysis of the transmission pathways is performed; this is a transformation process *in vivo*.^18,19^

The quality parameters of herbal drugs are usually specified and implemented in the respective pharmacopoeias. Selection of appropriate components for quality control is still a challenge, and the relationship between the extent and efficacy of drugs using some components at higher concentrations as indicators has been previously ignored. There is a certain supervision problem in relevant departments associated with the quality of traditional medicine products and quality standards. Moreover, due to the limitation of the technology, manpower, and material resources, many detection methods of components are not reasonable, and it is necessary to establish a multi-index evaluation system to strengthen the quality standards and related research and ensure the quality consistency.^20^ We hope to provide new ideas for quality research and standard establishment of traditional medicines and their products and overcome the shortcomings of existing quality standards through the emerging quality standard concept by modern methodology and technology.

## 3. Q-markers: a new concept on quality control

**Fig. 1:**
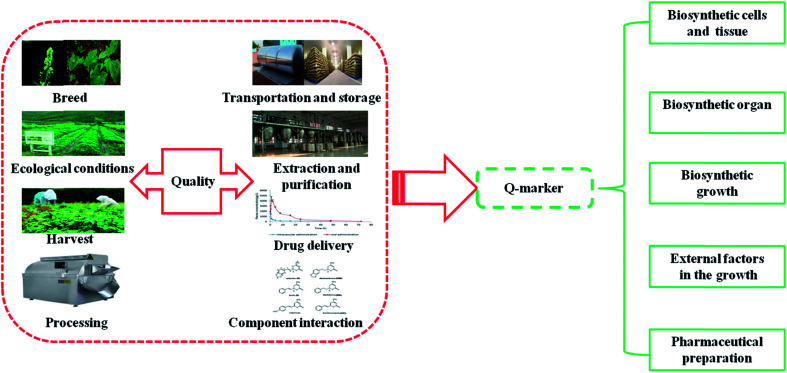
Main factors affecting the quality of traditional medicines and Q-marker.

As is commonly known, traditional medicines contain various chemical components and structural types, which are related to the effectiveness and safety of traditional medicines. Ingredients can be the quality markers of traditional medicine under certain conditions. Biosynthetic pathway of chemical ingredients is the basis of chemical biology and kinship. Compounds in plants switch molecular structure and obtain different biological activity for therapy by a variety of complex biosynthesis processes and synthesis mechanisms.^21,22^ A Q-marker, a new promising concept on quality control created by Liu, involves chemical substances from herbal medicine and products, such as tablets, decoctions, and extracts, that are closely related to the functional properties of traditional medicines that are inherent or formed in the process of preparation as markers of safety and effectiveness for quality control. It must also be mentioned that they are not chemical substances, such as metabolites, digestive enzymes or microbial transformed chemicals, absorbed by chemical processes and generated *in vivo*; thus, their chemical structures need to be determined, and quantitative and qualitative analysis should be conducted.^23^ Recently, studies have found that there are a large number of metabolic enzymes and substrates for synthesizing secondary metabolites in different plant cells and tissues.^24^ In specific organs composed of cell tissue, synthesis and accumulation of specific secondary metabolites show diversity in composition and content due to differences in related enzymes. Accumulation of secondary metabolites gives rise to cell differentiation and formation of plant organs, and consequently, secondary metabolites are accumulated in the plant organs.^25^ Multi-gene factors and environment interaction play a significant role in the formation process of secondary metabolites.^26,27^ Moreover, preparation technology is a remarkable factor that affects the quality of Q-markers, in which quality control is carried out based on the actual situation of various products to accurately establish the fingerprint of the sample and determine the bound of Q-marker.^28^ The core concept of Q-marker proposed for the traditional medicine system, integration of multidisciplinary knowledge, and the quality research of traditional medicine promote relevance of effectiveness, material basis, and quality control of marked ingredients, which focus on specificity, differences, dynamic changes of active ingredients, and quality transfer as well as traceability for contributing to establish quality control and quality traceability system of traditional medicines (Fig. 1).

## 4. Modern technology for identifying chemical components

With the development of modern science, advanced equipment, and technology, the method for identifying chemical components has achieved great improvement based on original traditional methods such as microscopic identification and physical or chemical identification. Selection of right appropriate research techniques is significant for the successful advancement of chemical component research and Q-marker determination.^29,30^ Currently, chromatography analysis, as a more conventional and effective identification method of traditional medicines, commonly includes paper chromatography, thin-layer chromatography, gas chromatography (GC), and high-performance liquid chromatography (HPLC) in actual identification.^31–33^ NMR and MS play crucial roles in the phases of component identification from novel chemical structures to Q-marker discovery in a high-throughput environment.^34^ MS, as an efficient technique for measuring the peak and relative intensities, exact mass, molecular formula, as well as relative error, has been attempted to manifest the chemical structure. MS identifies the category of chemical components based on their mass/charge ratio (*m*/*z*).^35,36^ By promoting the qualitative and quantitative analysis by LC or GC initially, chemical constituents are often infused directly into the mass spectrometer and then separated. GC-MS, as a highly sensitive, repeatable, and cost-efficient form of MS analysis method, requires time-consuming sample preparation that causing the sample changes during the operation. The advantage of LC-MS is that it requires a small amount of sample as compared to GC-MS in the overall preparation process.^37^ Chromatographic separation refers to two processes, *i.e.* dissolution of the sample in a solvent as the mobile phase and passing them through a stationary phase of specific interaction chemistries that allows a portion of the dissolved sample to remain in the stationary phase and a portion to pass at a different flow rate. Subsequently, the analyzed compounds are subjected to ionization using ESI, FAB, APCI, MALDI and others for the formation of mass separation fragments.^38,39^ Mass separation is performed by several mass analyzers such as time-of-flight (TOF), quadrupole, and ion trap, which possess different dynamic ranges, resolutions, and accuracies. Among them, the TOF mass analyzer, with high sensitivity and mass accuracy of the analysis, is suitable for structural analysis of unknown compounds.^40,41^

**Fig. 2:**
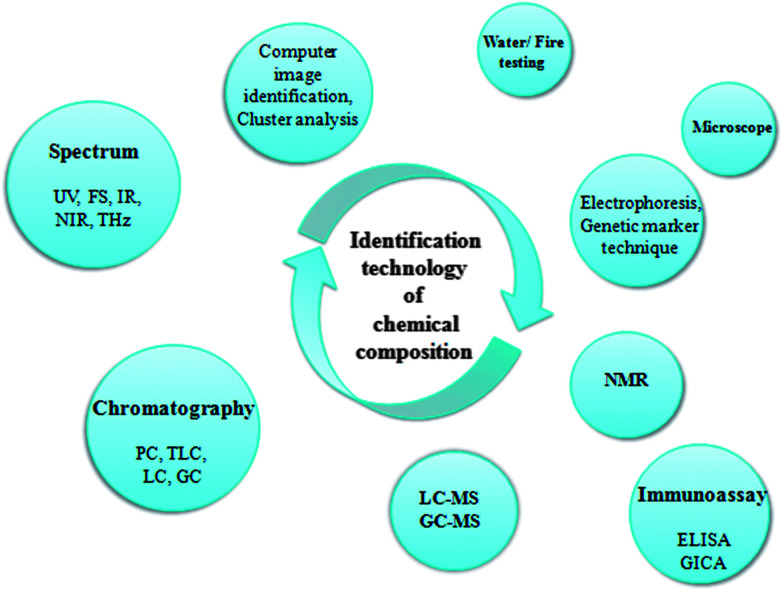
Main technology for identifying chemical components. UV, ultra violet; FS, fluorescence; IR, infrared; NIR, near-infrared; THz, terahertz; PC, paper chromatography; TLC, thin-layer chromatography; GC, gas chromatography; LC, liquid chromatography; MS, mass spectrum; NMR, nuclear magnetic resonance; ELISA, enzyme linked immunosorbent assay; and GICA, gold immuno-chromatography assay.

Analysis and identification of herbal medicines by spectrum has been accepted as an effective identification method by most countries, which identifies herbs by different wavelengths and penetrability of ultraviolet, fluorescence, and infrared radiation. After the discovery of the near-infrared (NIR) region in 1800, NIR spectroscopy sprung up, reformed in the early 1950s, and was used in the 1970s. Later, it has gradually matured and is now one of the most outstanding major analytical technologies based on the main characteristics of rapid measurement, no consumption detection, and direct qualitative identification and quantitative analysis in herbal products.^42,43^ Due to development in instrumentation, computing power, and multivariate data analysis, it has become the preferred quality control method in more applications and research after its first use in the cereal industry, which provides a more comprehensive and overall information of herbs.^44^ Instrument analysis possesses advantages including high sensitivity, high precision, good reproducibility, and standardization, but cannot meet the requirements of simple and rapid detection. Immunoassay, a qualitative and quantitative detection method of antigen or antibody, has the advantages of small sample dosage, simple operation, rapid detection, low cost, and high throughput and is based on the antigen and antibody specific reactions.^45^ At present, the most widely used immunoassay methods in the field of medicine research are enzyme-linked immunosorbent assay (ELISA) for high-throughput rapid quantitative detection and gold immuno-chromatography assay (GICA) for rapid detection of immune colloid test.^46,47^ Moreover, the identification technology of traditional medicines involves computer image identification, clustering analysis, electrophoresis, and DNA molecular genetic marker technology^48,49^ (Fig. 2).

## 5. Q-markers from herbs: new trends and effective strategies

### Based on quality transfer and traceability of composition

Since the chemical composition of herbs plays a role in the treatment of diseases through the process of synthesis in organisms, extraction and purification, production, absorption, and metabolism *in vivo*, it is important to pay attention to the abovementioned process of chemical composition based on the idea of the quality transmission and traceability for the establishment of the whole quality control system. *Eucommia ulmoides*, a type of herb dry bark, has the ability to enhance the immune response, possesses anti-ageing, anti-tumour, blood sugar reducing, blood lipid regulating, osteoporosis preventing, liver and kidney protecting, and other pharmacological properties in medical research.^50^ Upon comparing the herb extract group, administration group, and rat blank serum group through UHPLC-Q-TOF-MS technology, the results showed that the chemical constituents of five chromatographic peaks were determined using the control substance in the same retention time as the prototyping components, including teniposidic acid, protocatechuic acid, geniposide, pinoresinol diglucoside, and pinealol monoglucoside, which are direct active substances of *Eucommia ulmoides* and provide scientific basis for quality control.^51^*Radix polygalae* was first mentioned in Shen Nong Ben Cao Jing and dried roots of *Polygala tenuifolia* and *P. sibirica*, has been applied to relieve amnesia, neurasthenia, insomnia, nocturnal emission and palpitations.^52,53^ Ultra high-performance liquid chromatography associated with quadrupole time-of-flight mass spectrometry and the MarkerLynx TM software coupled with multiple data processing approaches was applied to select the compounds of *Radix polygalae in vitro* and *in vivo*. Typically, 35 compounds in the *Radix polygalae* are described, and 13 compounds in total absorbed into blood sample contain six prototype components such as sucrose, sibiricose A3, sibiricose A5, sibiricose A1, polygalaxanthone III, and *N*-acetyl-d-glucosamine, which narrow the range of screening the promising bioactive ingredients and offer a foundation for the quality control and exploration of action mechanism of *Radix polygalae*.^54^*Acanthopanax senticosus* has been widely used in folk medicinal herb for the treatment of chronic bronchitis, neurasthenia, hypertension, and ischemic heart disease. A fast and optimized UPLC method with Q-TOF mass spectrometry and the MarkerLynx™ software combined with multiple data processing approach has been developed for the analysis of constituents in leaves extracts and stem by comparison of *Acanthopanax senticosus* extracts and rat serum after administration. A total of 12 prototype components and 7 prototype components in animal blood derived from leaves and stem were identified or tentatively characterized as potential quality markers of *Acanthopanax senticosus*.^55,56^ The latest study found that geniposide in saffron and chemical ingredients, including chlorogenic acid, flavonoid glycosides, catechin, epicatechin, procyanidins, steroids, and triterpenoids, of the genus Cecropia were selected as chemical markers for quality control.^57,58^ A total of 337 chemical constituents were identified from the main raw materials of *Angelica dahurica*, Angelica, Ligusticum, Radix puerariae, Asarum, *Ligustrum lucidum*, and Schisandra oil in Liujing headache tablet by HPLC-Q/TOF-MS. The method of medicinal material BPI map is used for comprehensive analysis of chemical substance basis and searching the source of compounds, and then, 46 blood transitional components, including 24 prototype components and 22 metabolites, have been identified in the rat, in which the prototype components include isoflavones, phenanthrene components, phenylpropanoids, iridoid glycosides, phenylethanol glycoside, and other components.^59^ Huafeng Dan is composed of ox-gall water, rhizoma typhonii, raw pinellia, arisaema aridum, radix aconite, turmeric, basil leaves, batryticated silkworm, rhizoma gastrodiae, and other herbs, which is used for the treatment of rheumatism, stroke hemiplegia, epilepsy, and facial nerve paralysis embolism.^60^ UPLC-Q-TOF/MS was used to establish the fingerprints of Huafeng Dan and drug-containing serum of rats after intragastric administration of Huafeng Dan extract and the negative samples of Yaomu extracts for quantifying the changes in serum and its metabolism in the positive and negative ion detection modes, respectively. The result shows that five prototype components, *i.e.* Tarara auxiliaries, apigenin, rosmarinic acid, and two unknown new compounds, in rat serum originate from Yaomu, which has a dominant position in the prescription.^61^

### Based on biosynthetic pathway and specificity of composition

Corydalis rhizoma, mentioned in the Shen Nong's Herbal Classic, is applied for the treatment of abdominal pain, menstrual dysmenorrhea, postpartum stasis, and embolism pain where the alkaloid compounds in Corydalis are mainly divided into three species, namely protoberberine alkaloids, protopine alkaloids, and 7-oxo-aporphine alkaloids. There is no doubt that tyrosine is a common precursor in three types of alkaloids; however, higenamine and (*S*)-reticuline are two significant intermediates in the biosynthetic pathway. Under the catalytic action of specific synthetase, (*S*)-anonaine as the branching point of the synthesis pathway of tetrahydrophenylisoquinoline alkaloids can be further synthesized to D-sea papaverine, tetrahydropalmatine, berberine, protopine alkaloids, and other alkaloids. Compared with protoberberine alkaloids, protopine alkaloids and 7-oxo-aporphine alkaloids exist in the downstream pathway from the specificity analysis of components leading to tetrahydropalmatine, corydaline, coptisine, palmatine, deoxygenated corydaline, *D*-tetrahydroxylated alkaline, and protopine, considered as quality markers for Corydalis rhizoma.^62,63^*Swertia chirayita*, an endangered medicinal herb, is widely used as an antidiabetic in clinic treatment. It contains two major kinds of metabolites in the biosynthetic pathway named secoiridoids and xanthones such as swertiamarin, mangiferin, amarogentin, and amaroswerin. The study reports that LC-ESI-QTOF-HRMS/MS has been used to detect the missing intermediates for completing the biosynthetic pathway and amaronitidin biosynthesis requires coupled reaction of gentiopicroside and biphenyl acid derivatives such as amarogentin and amaroswerin. Gentiopicroside, mangiferin, amarogentin, iriflophenone, maclurin, deoxyloganic acid, loganic acid, and other intermediate metabolites are potential quality markers of chirayita.^64^ Polygonatum is a perennial herb mainly distributed in the northern region and northern subtropics, possessing anti-aging, immunity regulating, blood lipid regulating, memory improving, anti-tumor, and anti-bacterial properties along with other aspects of potential medicinal value.^65–67^ Due to their rich content in plants, polysaccharide and steroidal saponins were regarded as the main efficacy ingredients. At different oxidation levels, a number of polyoxygenated compounds were produced through oxidation reaction at different positions, which gave rise to molecular diversity of the steroidal saponins in polygonatum showing different levels of steroidal saponins oxidation and the diversification of the sugar chain structure. Genistein and diosgenin in the first-stage oxidation level are the precursors of the evolution of steroidal saponins in polygonatum. In addition, the research shows that glycosides of diosgenin are the common constituents of polygonatum, and saponin components can be used as evidence and feasible paths for the determination of quality markers of polygonatum in view of differences and specificity.^68^

### The correlation between chemical component and pharmacodynamics

A correlation analysis between the chemical fingerprints and efficacy evaluation was developed to identify quality marker components to assess herb quality. Gastrodia elata tuber (GET), as a famous herbal medicine, has been widely used in Asia for a long time. The study adopts a rational strategy of fingerprint–efficacy relationship based on HPLC coupled with QTOF-MS method and model of β-amyloid peptide (Aβ_25–35_)-induced PC12 cell death *in vitro* for exploring neuroprotective effects of the GET extracts. The result shows that 5-hydroxymethyl-2-furaldehyde (5-HMF), parishin B (PB), and parishin C (PC) were identified and regarded as quality markers of GET by interpreting the fingerprint–efficacy relationship of chemical fingerprints and neuroprotective effects using subsequent orthogonal projection to the latent structure-discriminant analysis (OPLS-DA) mode.^69^ Chemical fingerprints of 27 Rehmannia Glutinosa (RG) samples treating kidney yin deficiency and urinary metabolic profiling of RG treatment of kidney yin deficiency in rats were analyzed by LC-MS. Moreover, thirty-four variables in chemical fingerprints were successfully confirmed to have a close relationship with the efficacy of RG.^70^ As integral activity evaluation of herbal medicines for quality control has become more popular in recent years, spectrum-activity is easily ignored as compared to the relationship between chromatography/mass spectroscopy and bioactivity in most researches. Herein, six crucial markers, *i.e.* chlorogenic acid, 3,5-dicaffeoylquinic acid, 1,5-dicaffeoylquinic acid, luteoloside, apigenin-7-*O*-β-d-glucoside, and luteolin-7-*O*-6-malonylglucoside, were selected and identified from the near infrared reflection spectra (NIRS) of Flos Chrysanthemi samples and related anti-inflammation activities using the combination of HPLC/Q-TOF-MS identification, heat map clustering, boxplot analysis, and the interval limits of detection. The parameter optimization of these Q-markers in Flos Chrysanthemi powder has been developed by partial least squares regression (PLSR) calibration models associated with synergy interval partial least squares (siPLS) and spectral pretreatment methods, and then, a more excellent non-linear fitting effect is displayed *via* a back-propagation neural network (BP-ANN) after understanding the relationship between Q-marker contents and anti-inflammation activity, which is suitable for fast quality management in Flos Chrysanthemi and other herbs though the integrated NIRS and bioactive strategy.^71^ Serum chemical coupled with serum pharmacological was used to explore the material basis of Mahuang Fuzi Xixin decoction (MFXD) for anti-inflammation and immune-suppression. Herein, twenty-seven prototype components, including ten from Mahuang, thirteen from Fuzi, and four from Xixin, were discovered by comparison fingerprints of MFXD, drug-containing serum, and blank serum *via* LC-MS/MS. ELISA of histamine, β-hexosaminidase, and MTT for detecting the effect of drug-containing serum on lipopolysaccharide-induced splenocyte proliferation at different time points and a correlation analysis of components of MFXD and pharmacological indices were applied to explore the connection of material basis of MFXD, anti-inflammation, and immune suppression.^72^ Moreover, 10 alkaloids in the serum sample of Corydalis, 12 coumarin in the serum sample of Angelica, and 6 metabolites were selected for network pharmacology research according to the previous serum and pharmacological studies of Yuan Hu Zhixiao Pills, which showed that these 28 compounds have a close association with 52 targets and 31 pathways of dysmenorrhea.^73^ The results showed that all 28 compounds were able to act on the related pathways, reflecting the multi-component, multi-target mechanism of Yuan Hu Zhixie pills in the treatment of dysmenorrhea. The results showed that tetrahydropalmatine, palmatine, D-papaverine, protopanaxanthin, esperomycin, and isoepaproxen may be used to further modify the six active components of G-protein-coupled receptor binding assay. It is the basis of the main pharmacological substance of Yuan Hu Zhixiao pills, which can be used as a quality marker.^74^ In recent years, many scholars have established some new strategies for quality markers of herbs research on the basis of correlation of chemical components and their effects.

### Chinmedomics strategy

It is essential to establish high-throughput, rapid methods for screening and identifying bioactive constituents from herbs. Notably, only compounds in blood have the probability of becoming effective constituents. Disease as a functional state is caused by metabolic imbalances, which show different metabolite changes in the body from the perspective of modern medicine. Metabolomics offers a powerful way to establish metabolic profiling to discover metabolite biomarkers and related disease pathways.^75,76^ Correlation analysis between metabolite markers with constituents in serum originated from traditional medicines was performed for bioactive constituent discovery. Chinmedomics, a new platform for direct discovery and screening of highly correlated ingredients with the therapeutic effect of traditional medicines put forward by Professor Wang, was based on the combination of serum pharmacology and metabolomics for searching syndrome biomarkers, building evaluation system of drug efficacy, and then determining efficacy material.^77,78^*Phellodendri Amurensis Cortex* (PAC) as a well-known herbal medicine has been widely applied for immuno-suppression, antipyretic, anti-inflammatory, antibacterial, anticancer, anti-ulcer, antioxidant, anti-gout, blood pressure regulation, and many other pharmacological activities.^79,80^ Prostate cancer is one of the most common malignant tumours of the urogenital systems in European and American elderly men. Global constituents and serum metabolites of PAC were detected, and the inhibiting effects of prostate cancer were evaluated though UPLC-MS on the basis of chinmedomics analysis method, which indicated that 54 peaks in the spectrum of PAC were characterized *in vitro* and 38 peaks were characterized *in vivo*. Moreover, 29 prototype components were absorbed into the serum, and the others were metabolites among the 38 compounds. In addition, thirty-four metabolic biomarkers associated with linoleic acid metabolism, arachidonic acid metabolism, sphingolipid metabolism, glycerophospholipid metabolism, alpha-linolenic acid metabolism, retinol metabolism, glyoxylate and dicarboxylate metabolism, and other metabolisms in organisms are closely related to prostate cancer, and PAC can observably regulate the disturbed metabolic profile to a healthy state. Furthermore, ten absorbed effective compounds were related with the therapeutic effect of PAC chinmedomics approach.^81^ In addition, we screened other active ingredients of herbs and products by chinmedomics for establishing a good foundation of quality marker selection.^82,83^

### Property–response–component mode

There are many problems in the biological model method based on the binary research of the component-effect, which neglects the basic attributes of medicinal properties of traditional medicines leading to incomplete description of medicine function and cannot properly build the correlation between the basic attributes of medicinal properties and the core content of traditional medicines theory. Zhang *et al.* put forward a ternary mode of property–response–component for studying effective material, basics, and mechanism of traditional medicines that illuminates effective components though identification of chemical substances prototype and achievement and confirmation of chemical structure and components absorbed in blood. Material basis of drug properties was screened and determined from gustation (olfactory) feature and function in terms of docking screening strategy based on the gustation bionic mode combined with gestation as well as olfactory receptor molecular and G-protein coupled receptor test. Network pharmacology, metabonomics and pharmacokinetics methods were used to explain the effect and mechanism of traditional medicines. According to the property–response–component theory, corydaline, tetrahydropalmatine, protopine, imperatorin, and isoimperatorin were determined to be the Q-markers of Yuanhu Zhitong dropping pills as an example of demonstration research.^84^

### Efficacy–syndrome–toxicity mode

In previous research, the evaluation of the quality of traditional medicines based on efficacy–toxicity mode for material basis confirmation has been carried out for a long time, but the relationship between the expression of efficacy with toxicity and syndrome has been neglected. The establishment of animal syndrome model for the evaluation of efficacy and toxicity correlation and syndrome in objectifying and scientization is a prerequisite for further study of Q-marker in the context of efficacy–syndrome–toxicity theory proposed by Professor Sun Rong. As the main chemical and bioactive components of Evodia rutaecarpa, alkaloids, especially evodiamine, rutaecarpin, and dictamnolactone are the active ingredients. After processing of Evodiae Fructus, the contents of evodiamine, rutaecarpin, and dictamnolactone were decreased but not obviously, and the content of limonin was higher, which suggested that limonin might be the basis of toxic substances. Using the network pharmacological analysis to find the pathways associated with analgesic, anti-inflammatory, and oxidative damage, the relevant target pathway prediction of four representative compounds, *i.e.* evodiamine, rutaecarpin, dictamnolactone, and β-pinene, by Cytoscape 2.6 software show that 32 targets and 5 pathways associated with abirritation of Evodia rutaecarpa and hepatic toxicity reflect the multi-component, multi-target, and multi-channel mechanism of the quality control of Evodia rutaecarpa on efficacy–toxicity network regulation.^85,86^

### Effect-constituent index

Based on the chemical analysis and bioeffect detection, some researchers propose a comprehensive evaluation index, effect-constituent index (ECI), for offering significant references to the QC of herbs and better service in clinic by enriching many of the efficacy-oriented quality control model studies of herbs.^87,88^ It is mainly carried out for the relatively clear efficacy and active ingredients of herbs.^89,90^ ECI fuses the accuracy of chemical composition detection and the advantages of technological safety related to drug efficacy, which greatly enhances the quality evaluation of traditional medicines in terms of accessibility and universality and resolves the puzzle by assessing the contribution of different components in the whole drug. At present, ECI has been developed and promoted in herbal medicines such as *Salvia miltiorrhiza*, *Rhubarb*, *Coptis chinensis*, and *Aconitum carmichaelii*.^91–94^ The activity data obtained from an antiplatelet aggregation test show significant variations in the contents of nine main constituents, including cryptotanshinone and salvianolic acid B in *Salvia miltiorrhiza* (SM), do not have good associations (*r* < 0.81) with the biopotency of SM itself. To calculate ECI, which is the sum of the products of content (*C*_i_) and biopotency weight for each constituent, cryptotanshinone and five other constituents of high biopotency were selected as quality-markers. Compared to the chemical content, ECI either had the highest correlation coefficient with the biopotency of SM or the lowest residual by regression analysis.^95^ Some studies proposed a toxicity calibrated content determination method for hyper toxic aconite, called toxic constituent index (TCI). Different batches of aconite were selected, and their evaluation results of toxic potency (TP), diester diterpenoid alkaloids (DDAs), and TCI were compared after measuring the minimum lethal dose value of mesaconitine (MA), aconitine (AC), and hypaconitine (HA) and establishing the TCI equation. By linear regression analysis and prediction error value study, TCI and TP manifest the highest correlation relevance, and testified that TCI was an simple and easy operation evaluation approach for toxicity prediction and quality control.^96,97^

### Based on drug properties of herbs

Drug properties are essential features of traditional medicine effectiveness and are an important basis of treatment and prescription in clinics. In some reports, the method of material basis of pungent-tasting herbs was used by an electronic nose and electronic tongue to describe the smell and the pungent taste of herbs, material group, and simplex components. After principal component analysis (PCA) of five pungent-tasting herb data obtained with tongue and nose, it was shown that different samples were obviously distinguished by an electronic nose with a high discrimination index, and the different taste samples distributed at different positions; this indicated that the electronic nose and electronic tongue had the ability to differentiate between the smell and taste of the material group with PCA and function.^98^ G protein-coupled receptors, a large class of membrane protein receptors, are referred to a number of diseases, and about 40% of modern drugs are targeted at G protein-coupled receptors. The chemical constituents of 60% ethanol extract obtained from Corydalis rhizoma were identified through HPLC-QTOF/MS. Using the fluorescence assay of intracellular calcium ion, stimulation and inhibition effects of Corydalis rhizoma and its representative compounds, such as protopine, palmatine, and tetrahydropalmatine, on G protein-coupled receptor closely related with drug properties, and 5-hydroxytryptamine 1A receptor (5-HT_1A_), μ opioid receptor (OPRM1), β_2_ adrenergic receptor (ADRB2), dopamine receptor (D_2_), acetylcholine receptor (M_2_), and thromboxane-prostaglandin receptor (TP) were detected. A total of 31 compounds were obtained and 28 alkaloid compounds were confirmed. In GPCR experiments, it was shown that Corydalis rhizoma could activate 5-HT_1A_, OPRM1, and ADRB2 receptors and inhibit D_2_ receptor. Protopine exerted confrontation on D_2_ and M_2_ receptors and tetrahydropalmatine was capable of agitating the ADRB2 receptor and restrained the activity of D_2_ and TP receptors; however, palmatine demonstrated no significant biological effect on the 6 G protein-coupled receptor.^99^

### Testability method of component and specificity

**Fig. 3:**
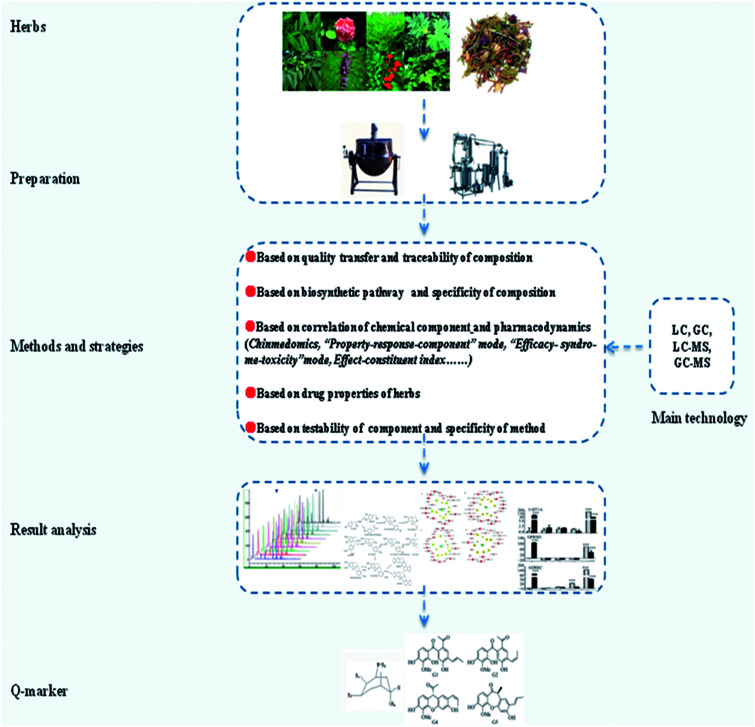
The main stages of screening Q-markers.

Most of the traditional medicine achieves qualitative identification and quantitative determination by chromatography and related technology; thus, the testability Q-marker in the chromatography is very important due to ease of operation and simple handling, which is difficult to realize for specific and active ingredients. It is still difficult to overcome some issues such as slightly less content, complicated component, and hardly any separation in herbs although increased means of detection can be selected. The study has found that the compound Y14 in *Leonurus japonicus* and compound G2 in *Penthorum chinense* can be detected and separated in consideration of strong specificity and clear structure and closely related with the functional properties. In the process of preparing the test solution of herbs, other components of *Penthorum chinense* had a great influence on the chromatographic behavior of the G2. Hence, the polyamide column was used to pretreat the 60% methanol extract and then obtain 70% ethanol elution for the preparation of TLC and HPLC test solution. This is the first time that the qualitative identification and quantitative determination of lignins in *Penthorum chinense* and the preparation of *Leonurus japonicus* test solution without chromatographic pretreatment can be well separated and detected.^100^ After the oral administration of Polygonati rhizoma, most steroidal saponins were hydrolyzed into secondary glycosides or aglycones to be absorbed into blood for the biological effects. Given this, total saponins, different types of aglycone or characteristic steroidal saponins were regarded as quality control indices, and the detection of steroid saponin without a conjugated system and UV absorption can be applied using an evaporative light-scattering detector^101^ (Fig. 3).

### Others

The traditional Chinese medicine system pharmacology database and analysis platform (TCMSP), a database for system pharmacology, is established based on system pharmacology for herbal medicines, which offers twelve vital properties about ADME and also gives out drug targets and human diseases of active ingredients designed to promote integration and development of traditional medicine.^102^ Moreover, cancer HSP is an anticancer herb database of systems pharmacology, which contributes towards the exploration of the molecular mechanisms of natural anticancer products and promotes anticancer drug development by manual curation of records of anticancer herbs associated with information.^103^ It was reported that weighted ensemble similarity (WES) was a new algorithm for identifying drug direct targets by predicting direct or indirect interactions on a small scale.^104^ Some researchers used system-based analysis to introduce a drug-target-pathway-organ network, which illuminated mechanisms of various diseases treated by the same strategy.^105^

## 6. Conclusion and future perspective

As a crucial part of traditional medicines, herbs have been used for medicinal purposes for centuries, which have been accepted by the public in food production to improve the taste and add trace elements for body and food preservation and in medicine to treat various diseases. Quality evaluation and control of traditional medicines have always been challenging in medicine research and are one of the key scientific problems that restrict the modernization of traditional medicines. Currently, the quality control mode is single, resulting in difficulty to correspond with the effect, which cannot control quality in the true sense. Quality control of traditional medicines needs a comprehensive, systematic, effective, and controllable standard to incarnate the quality and clinical efficacy. Q-marker is a new concept that has been put forward under the modern scientific and technological conditions for meeting quality control requirements of traditional medicines with high basic requirements such as deep fundamental research on pharmacological substances, secondary metabolites with specificity and biological activity, and better chromatographic behavior identified and determined under the current technical conditions. Therefore, for the Q-marker study of herbs, a systematic study is essential to support it by investing a lot of basic research in the establishment of multi-angle comprehensive evaluation methods and continuing to improve the screening identification technology. Although Q-marker research has a long way to go for a large number of herbs, the research and application of herbal Q-marker is still expected to endlessly make progress with the help of rapidly evolving science and technology for improving the quality control system of traditional medicines and promoting the modernization and development of medicine.

## Competing financial interests

The authors declare no competing financial interests.

## Conflicts of interest

There are no conflicts to declare.

## Supplementary Material
